# Significance of anti-neutrophil cytoplasmic antibodies in systemic sclerosis

**DOI:** 10.1186/s13075-019-1839-5

**Published:** 2019-02-14

**Authors:** Jayne Moxey, Molla Huq, Susanna Proudman, Joanne Sahhar, Gene-Siew Ngian, Jenny Walker, Gemma Strickland, Michelle Wilson, Laura Ross, Gabor Major, Janet Roddy, Wendy Stevens, Mandana Nikpour

**Affiliations:** 1 The University of Melbourne, 41 Victoria Parade Fitzroy, Melbourne, Victoria 3065 Australia; 2 St. Vincent’s Hospital Melbourne, 41 Victoria Parade Fitzroy, Melbourne, Victoria 3065 Australia; 3 University of Adelaide, Adelaide, South Australia Australia; 4 Royal Adelaide Hospital, Adelaide, South Australia Australia; 5 Monash Health, Melbourne, Victoria Australia; 6 Monash University, Melbourne, Victoria Australia; 7 Flinders Medical Centre, Adelaide, South Australia Australia; 8 Barwon Rheumatology Service, Geelong, Victoria Australia; 9 Royal Newcastle Centre John Hunter Hospital, Newcastle, New South Wales Australia; 10 University of Newcastle, Newcastle, New South Wales Australia; 11 Fiona Stanley Hospital, Perth, Western Australia Australia

**Keywords:** Anti-neutrophil cytoplasmic antibodies (ANCA), Systemic sclerosis (SSc), ANCA-associated vasculitis, Myeloperoxidase (MPO), Proteinase-3 (PR3)

## Abstract

**Background:**

Up to 12% of patients with systemic sclerosis (SSc) have anti-neutrophil cytoplasmic antibodies (ANCA). However, the majority of these patients do not manifest ANCA-associated vasculitis (AAV) and the significance of ANCA in these patients is unclear. The aim of this study is to determine the prevalence of ANCA in a well-characterised SSc cohort and to examine the association between ANCA and SSc clinical characteristics, other autoantibodies, treatments and mortality.

**Methods:**

Clinical data were obtained from 5 centres in the Australian Scleroderma Cohort Study (ASCS). ANCA positive was defined as the presence of any one or combination of cytoplasmic ANCA (c-ANCA), perinuclear ANCA (p-ANCA), atypical ANCA, anti-myeloperoxidase (anti-MPO) or anti-proteinase-3 (anti-PR3). Associations of demographic and clinical features with ANCA were investigated by logistic or linear regression. Survival analysis was performed using Kaplan-Meyer curves and Cox regression models.

**Results:**

Of 1303 patients, 116 (8.9%) were ANCA positive. Anti-PR3 was more common than anti-MPO (13.8% and 11.2% of ANCA-positive patients, respectively). Only 3 ANCA-positive patients had AAV. Anti-Scl-70 was more common in ANCA positive vs ANCA negative (25% vs 12.8%, *p* < 0.001), anti-MPO positive vs anti-MPO negative (38.5% vs 13.6%, *p* = 0.006) and anti-PR3 positive vs anti-PR3 negative patients (44.4% vs 13.4%, *p* < 0.001). A higher prevalence of interstitial lung disease (ILD) was found in the ANCA positive (44.8% vs 21.8%, *p* < 0.001) and the anti-PR3 positive groups (50.0% vs 23.4%, *p* = 0.009). In multivariable analysis, ANCA-positive status remained associated with ILD after adjusting for anti-Scl-70 antibodies. Pulmonary embolism (PE) was more common in ANCA-positive patients (8.6% vs 3.0%, *p* = 0.002) and anti-PR3-positive patients (16.7% vs 3.3%, *p* = 0.022). ANCA-positive status remained associated with PE in a multivariable analysis adjusting for anti-phospholipid antibodies. Kaplan-Meier analysis revealed increased mortality in ANCA-positive patients (*p* = 0.006). In Cox regression analysis, ANCA was associated with increased mortality, after adjusting for age and sex.

**Conclusions:**

ANCA is associated with increased prevalence of ILD and PE in SSc. ANCA should be tested in SSc, as it identifies individuals with worse prognosis who require close monitoring for adverse outcomes.

## Background

Systemic sclerosis (SSc) is a multisystem autoimmune disease characterised by a triad of progressive skin and internal organ fibrosis, autoantibody production and small vessel vasculopathy [1, 2]. Clinically, SSc patients are classified as having limited (lcSSc) or diffuse (dcSSc) SSc, defined by the extent of skin thickening [3].

Abnormal autoantibody production is a characteristic feature of SSc, and the presence of specific autoantibodies is of prognostic and clinical significance. Anti-centromere antibodies are classically associated with lcSSc and pulmonary arterial hypertension (PAH), while anti-Scl-70 antibodies are more frequently observed in dcSSc and interstitial lung disease (ILD) [4].

Anti-neutrophil cytoplasmic antibodies (ANCA) are autoantibodies directed against enzymes found within primary granules of neutrophils and lysosomes in monocytes and are implicated directly in the pathogenesis of the small vessel vasculitis [5, 6]. There are three different types of ANCA, which display distinct patterns under indirect immunofluorescence (IIF). These are the cytoplasmic pattern (c-ANCA), perinuclear pattern (p-ANCA) and “atypical” ANCA [5]. c-ANCA directed against proteinase-3 (PR3) is associated with granulomatosis with polyangiitis (GPA), while p-ANCA is typically directed against myeloperoxidase (MPO) and is commonly detected in microscopic polyangiitis (MPA), eosinophilic granulomatosis with polyangiitis (EGPA) and pauci-immune idiopathic crescentic glomerulonephritis [7]. In addition, p-ANCA can be directed against antigens other than MPO and is observed in a variety of autoimmune disorders including rheumatoid arthritis (RA), systemic lupus erythematosus (SLE), Sjogren’s syndrome (SS), polymyositis (PM) and dermatomyositis (DM) [8]. Alternative target antigens for p-ANCA include elastase, lactoferrin, bactericidal/permeability-increasing protein (BPI) and cathepsin G, and the pathophysiological significance, and specific disease associations of these minor target antigens are unclear [8, 9].

The presence of ANCA in the sera of patients with SSc is relatively uncommon, with previous studies estimating a prevalence between 0 and 12% [7, 10, 11]. Despite up to 12% of SSc patients having a positive ANCA titre, only a minority of these patients will develop an overlap syndrome with AAV. A 2013 review found a total of only 51 cases of AAV in SSc published in the literature [10]. A study of 2200 SSc patients found only 8 patients (0.4%) with comorbid AAV and SSc [12]. The majority of published cases of AAV and SSc overlap describe MPA or renal-limited vasculitis [10]. Anti-MPO and p-ANCA are most commonly found, and c-ANCA positivity and GPA are rarely reported [13].

The underlying features of SSc that are associated with ANCA and AAV are contentious. AAV has been described in both lcSSc and dcSSc, with conflicting reports as to which SSc disease subtype has a higher prevalence of AAV [13, 14]. Anti-Scl-70 antibodies have been frequently associated with the development of AAV, with reports of up to 77% of SSc patients with AAV having anti-Scl-70 antibodies [10, 13, 15]. SSc patients who have overlap syndromes with other connective tissue diseases may have an increased prevalence of AAV. Case series have suggested up to 50% of SSc patients with AAV also have clinical features of other connective tissue diseases such as SLE [14].

Another consideration is the possibility of drug-induced ANCA or AAV in SSc. Although no longer part of the therapeutic armamentarium in SSc, d-penicillamine has been associated with the development of ANCA-associated glomerulonephritis (AAGN) and some cases of AAGN in SSc may be reflective of prior d-penicillamine therapy [10, 16–19]. However, it is difficult to ascertain whether d-penicillamine therapy or SSc itself is the underlying cause of ANCA production, particularly in cases where AAGN occurs several years after cessation of therapy [15, 19].

The clinical significance of ANCA in SSc patients who do not manifest AAV is controversial. An association between ANCA in SSc and ILD has been suggested [12, 20–22]. However, this has not been consistently reported in all case series [15]. It has also been suggested that ANCA in SSc patients may indicate an inflammatory component to the illness and that ANCA should be treated as a “red flag”, prompting a thorough investigation and follow-up [23]. In the present study, we examined the clinical significance of ANCA in a large, well-characterised SSc cohort, including the association between ANCA and SSc clinical characteristics, autoantibodies, treatments and mortality.

## Methods

### Patients

Patients were recruited from the Australian Scleroderma Cohort Study (ASCS), a multicentre study of risk and prognostic factors in SSc across 5 participating Australian centres (St. Vincent’s Hospital, Melbourne and Monash Health, Victoria; John Hunter Hospital, New South Wales; Royal Adelaide Hospital, South Australia; Fiona Stanley Hospital, Western Australia). All human research ethics committees of the participating sites have approved the ASCS. Written informed consent was obtained from all patients at recruitment.

### Inclusion and exclusion criteria

We included patients from the ASCS recruited between January 1, 2007, and May 23, 2016, who fulfilled the 2013 American College of Rheumatology (ACR)/European League Against Rheumatism (EULAR) criteria [24] for diagnosis of SSc. Patients with mixed connective tissue disease were excluded from the study.

ANCA testing is routinely performed as part of a panel of tests requested at entry to the ASCS. Patients were excluded if they did not have an ANCA result recorded in the database. All positive ANCA results were confirmed by inspection of the original laboratory results. Patients were excluded if original results were not available for review. For statistical analyses involving autoantibodies, patients without a documented autoantibody result were excluded from that particular analysis.

### ANCA testing

The presence of ANCA was defined by a positive result for any one or a combination of p-ANCA, c-ANCA, atypical ANCA, anti-MPO and anti-PR3. Indirect immunofluorescence (IIF) was performed using the local laboratory protocol to detect p-ANCA, c-ANCA and atypical ANCA. IIF results described as “unable to exclude p-ANCA due to the presence of ANA” were defined as ANCA negative if anti-MPO or anti-PR3 were negative. Anti-MPO and anti-PR3 were measured by enzyme-linked immunosorbent assay (ELISA) using the local laboratory commercial test kit and reference range.

For each ANCA-positive patient, a retrospective case note review was performed to assess for clinical and histopathological evidence of vasculitis. Cases of ANCA-negative vasculitis were not explored in this study.

For statistical analysis, patients were divided into categories based on their ANCA results. Demographics and clinical manifestations were compared between ANCA-positive patients and ANCA-negative patients, anti-MPO-positive patients and anti-MPO-negative patients, and anti-PR3 positive patients and anti-PR3 negative patients.

### Data collection

Demographic and disease data were prospectively collected at baseline and at subsequent annual reviews as per a standardised protocol. All disease features and autoantibodies were defined as present if they were ever present from the time of diagnosis. Disease onset and disease duration were defined from the date of onset of the first non-Raynaud’s manifestation. Disease subtype was defined as diffuse or limited as per LeRoy criteria [25]. Patients were diagnosed with SSc overlap syndrome at physician discretion if features of RA, SLE, SS, PM or DM were clinically evident. Anti-nuclear antibody (ANA) was detected by indirect immunofluorescence (IIF) using the local laboratory protocol. Extractable nuclear antigen (ENA) testing was performed by ELISA, immunoblot or a combination of these two methods, using the local laboratory commercial test kits. Anti-dsDNA testing was performed by ELISA in most laboratories, with two laboratories using the Farr radioimmunoassay. ILD was diagnosed on high-resolution-computed tomography (HRCT) of the chest, which was performed on the basis of abnormal respiratory function tests or the presence of crepitations on respiratory system examination. Pulmonary arterial hypertension (PAH) was defined by right heart catheterisation as a mean pulmonary artery pressure ≥ 25 mmHg and a pulmonary arterial wedge pressure ≤ 15 mmHg. Scleroderma renal crisis was defined by a combination of any two of three criteria, which include new-onset hypertension in the absence of an alternate aetiology, rising creatinine or microangiopathic haemolytic anaemia. Small intestinal bacterial overgrowth (SIBO) was considered present if a patient described concurrent diarrhoea and the use of cyclical antibiotics. Gastric antral vascular ectasia (GAVE), reflux oesophagitis and oesophageal stricture were defined on endoscopy. Data regarding hospitalisations were collected annually from patient-reported admissions for any reason, for a period of greater than 24 h. Patients were recorded as having had a malignancy if they reported the presence of any skin, solid organ or haematological malignancy. Treatments were defined as use ever from disease onset to the most recent visit. The use of biologics included patients ever being exposed to any of rituximab, abatacept, tumour necrosis factor (TNF) alpha inhibitors and tocilizumab.

### Statistical analysis

Data are presented as the mean ± standard deviation for continuous variables and as a number (percentage) for categorical variables. Univariable analyses of the relationship between positive ANCA, and positive anti-MPO and anti-PR3 were conducted using the chi-squared or Yate’s corrected chi-squared/Fisher’s exact test (where appropriate) for categorical variables. The *t* test or rank sum test (where appropriate) was utilised for continuous variables. Summary statistics, univariable and multivariable logistic regression were performed to determine the correlates of ANCA. Multicollinearity and first-order interaction between variables were taken into consideration when selecting variables for inclusion in the regression models. Kaplan-Meier (KM) survival graphs and Cox proportional hazards regression analysis were used to compare survival between the ANCA-positive and ANCA-negative groups. Statistical significance was defined as *p* ≤ 0.05. All statistical analyses were performed using Stata/SE 15.1 software (StataCorp, College Station, TX, USA).

## Results

### Study population

A total of 1303 patients fulfilled the inclusion criteria for this study, and the characteristics of this cohort are summarised in Table 1. Of the 1303 patients included in the study, 1125 (86.3%) were females, 974 (74.8%) had limited disease and 329 (25.2%) had diffuse disease. The majority of patients were Caucasian (90.4%) followed by Asian (4.5%). The mean ± standard deviations (SD) of the age of SSc onset and the age at recruitment were 46.4 ± 14.3 and 57.7 ± 12.5 years, respectively. The median ± interquartile range (IQR) duration of follow-up was 3.46 ± 2.69 years. Almost half (49%) of the cohort had ever smoked.

**Table 1:** Cohort characteristics (*n* = 1303)

Characteristic	Values
Female	1125 (86.3%)
Race	
Caucasian	1178 (90.4%)
Asian	59 (4.5%)
Aboriginal-Islander	15 (1.2%)
Other	18 (1.4%)
Disease subtype	
Limited	974 (74.8%)
Diffuse	329 (25.2%)
Age at scleroderma onset, years	46.40 ± 14.26
Age at recruitment, years	57.71 ± 12.54
Disease duration at recruitment, years	11.26 ± 10.31
Duration of follow-up, years	3.46 ± 2.69
Ever smoked	639 (49.0%)
Autoantibodies	
ANCA	116 (8.9%)
Anti-Scl-70	181 (13.9%)
Anti-centromere	613 (47.0%)
Antinuclear antibody	1222 (93.8%)
Rheumatoid factor	337 (25.9%)
Anti-Ro	87 (6.7%)
Anti-La	20 (1.5%)
Anti-RNP	26 (2.0%)
Anti-dsDNA	42 (3.2%)
Anti-Sm	8 (0.6%)
Anti-Jo-1	7 (0.5%)
Anti-Scl/PM	15 (1.2%)
Anti-cardiolipin	262 (20.1%)
Anti-beta 2 glycoprotein	94 (7.2%)
Lupus anti-coagulant	35 (2.7%)
Anti-RNA polymerase	116 (8.9%)
Clinical characteristics and complications	
Pulmonary arterial hypertension	163 (12.5%)
Interstitial lung disease	311 (23.9%)
Renal crisis	35 (2.7%)
Digital ulcers	646 (49.6%)
SIBO	37 (2.8%)
GAVE	112 (8.6%)
Oesophageal dysmotility	132 (10.1%)
Oesphageal stricture	146 (11.2%)
Synovitis	517 (39.7%)
Malignancy	252 (19.3%)
Hospitalisation	490 (37.6%)
Deep vein thrombosis (DVT)	91 (7.0%)
Pulmonary embolus (PE)	46 (3.5%)
Overlap features with another connective tissue disease	76 (5.8%)
Overlap syndrome with rheumatoid arthritis	27 (2.1%)
Overlap syndrome with polymyositis	13 (1.0%)
Overlap syndrome with Sjogren’s	24 (1.8%)
Overlap syndrome with SLE	12 (0.9%)
Overlap syndrome with dermatomyositis	1 (0.1%)
Treatments	
Prednisolone	580 (44.5%)
Azathioprine	105 (8.1%)
Mycophenolate	103 (7.9%)
Cyclophosphamide	118 (9.1%)
Calcium channel antagonist	847 (65.0%)
Topical vasodilator	105 (8.1%)
Iloprost	161 (12.4%)
Penicillamine	108 (8.3%)
Rituximab	11 (0.8%)
Abatacept	1 (0.1%)
TNF alpha inhibitor	11 (0.8%)
Tocilizumab	5 (0.4%)
Biologics	26 (2.0%)

Values are given as number (%), mean ± SD or median (IQR)

*Abbreviations: ANA* anti nuclear antibodies, *ANCA* anti-neutrophil cytoplasmic antibodies, anti-phospholipid antibodies: any one or combination of anti-cardiolipin, anti-beta-2-glycoprotein and lupus anticoagulant, *anti-dsDNA* anti-double stranded deoxyribonucleic acid, *anti-RNP* ribonucleoprotein, *SIBO* small intestinal bacterial overgrowth, *SLE* systemic lupus erythematosus, *anti-Sm* anti-Smith

The autoantibody profile of the cohort is summarised in Table 1. Overall, 93.8% were anti-nuclear antibody (ANA) positive, 47.0% were anti-centromere positive and 13.9% were anti-Scl-70 positive. Other autoantibodies with significant prevalence within the cohort were rheumatoid factor (RF) (25.9%), anti-cardiolipin (20.1%), RNA polymerase (8.9%) and anti-Ro (6.7%).

Disease features of the cohort are summarised in Table 1. ILD was present in 311 patients (23.9%), and 163 patients (12.5%) had PAH. Almost half (49.6%) of patients had digital ulcers, 39.7% had synovitis, 8.6% had GAVE and 2.7% had a renal crisis. Overlap features with another connective tissue disease were present in 5.8%, and comorbid rheumatoid arthritis was the most common overlap syndrome, present in 27 patients (2.1%).

Treatment characteristics of the cohort are summarised in Table 1. A significant proportion of the cohort had received calcium channel antagonists (65.0%) and prednisolone (44.5%). With respect to immunosuppressive therapies, 8.1% of patients received azathioprine, 7.9% had received mycophenolate and 9.1% had received cyclophosphamide. Only 26 patients (2.0%) had received biologics.

### ANCA-positive vs ANCA-negative groups

Of the cohort of 1303 patients, 116 patients (8.9%) were ANCA positive, and 1187 (91.1%) were ANCA negative. Univariable analyses comparing demographic characteristics between the ANCA-positive and ANCA-negative groups are summarised in Table 2.

**Table 2:** Characteristics of ANCA-positive and ANCA-negative patients (*n* = 1303)

	ANCA negative *n* = 1187	ANCA positive *n* = 116	*p*
Female	1028 (86.6%)	97 (83.6%)	0.372
Race			
Caucasian	1082 (91.2%)	96 (82.8%)	
Asian	44 (3.7%)	15 (12.9%)	0.001^#^
Aboriginal-Islander	14 (1.2%)	1 (0.9%)	
Other	17 (1.4%)	1 (0.9%)	
Type of disease			
Limited	890 (75.0%)	84 (72.4%)	
Diffuse	297 (25.0%)	32 (27.6%)	0.544
Age at scleroderma onset, years	46.43 ± 14.16	45.95 ± 15.74	0.744
Age at recruitment, years	57.73 ± 12.31	57.54 ± 14.72	0.880
Disease duration at recruitment, years	8.29 (13.27)	9.06 (13.84)	0.891^^^
Duration of follow-up, years	3.07 (4.54)	3.48 (3.48)	0.174^^^
Ever smoked	597 (50.3%)	42 (36.2%)	0.004
Autoantibodies			
Anti-Scl 70	152 (12.8%)	29 (25.0%)	< 0.001
Anti-centromere	579 (48.8%)	34 (29.3%)	< 0.001
ANA	1115 (93.9%)	107 (92.2%)	0.360
Rheumatoid factor	301 (26.7%)	36 (31.0%)	0.313
Anti-Ro	74 (6.2%)	13 (11.2%)	0.040
Anti-La	17 (1.4%)	3 (2.6%)	0.413^#^
Anti-RNP	24 (2.0%)	2 (1.7%)	> 0.999^#^
Anti-dsDNA	34 (2.9%)	8 (6.9%)	0.062
Anti-Sm	7 (0.6%)	1 (0.9%)	0.527^#^
Anti-Jo-1	6 (0.5%)	1 (0.9%)	0.480^#^
Anti-Scl/PM	14 (1.2%)	1 (0.9%)	> 0.999^#^
Anti-cardiolipin	231 (19.5%)	30 (25.9%)	0.801^#^
Anti-Beta 2 glycoprotein	83 (7.0%)	11 (9.5%)	0.658
Lupus anticoagulant	31 (2.6%)	4 (3.4%)	0.778^#^
Anti-RNA polymerase	105 (8.8%)	11 (9.5%)	0.409
Clinical characteristics and complications			
Pulmonary arterial hypertension	107 (9.0%)	15 (12.9%)	0.167
Interstitial lung disease	259 (21.8%)	52 (44.8%)	< 0.001
Renal crisis	32 (2.7%)	3 (2.6%)	> 0.999^#^
Digital ulcers	587 (49.5%)	59 (50.9%)	0.772
SIBO	35 (2.9%)	2 (1.7%)	0.767^#^
GAVE	107 (9.0%)	5 (4.3%)	0.085
Oesophageal dysmotility	124 (10.4%)	8 (6.9%)	0.226
Oesphageal stricture	137 (11.5%)	9 (7.8%)	0.218
Synovitis	454 (38.2%)	63 (54.3%)	0.001
Malignancy	221 (18.6%)	31 (26.7%)	0.035
Hospitalised	433 (36.5%)	57 (49.1%)	0.007
Deep vein thrombosis (DVT)	83 (7.0%)	8 (6.9%)	0.969
Pulmonary embolus (PE)	36 (3.0%)	10 (8.6%)	0.002
Overlap features with another connective tissue disease	62 (5.2%)	14 (12.1%)	0.003
Overlap syndrome with rheumatoid arthritis	22 (1.9%)	5 (4.3%)	0.076
Overlap syndrome with polymyositis	12 (1.0%)	1 (0.9%)	> 0.999^#^
Overlap syndrome with Sjogren’s	19 (1.6%)	5 (4.3%)	0.038
Overlap syndrome with SLE	9 (0.8%)	3 (2.6%)	0.084^#^
Overlap syndrome with dermatomyositis	0 (0.0%)	1 (0.9%)	0.089^#^
Treatments			
Prednisolone	520 (43.8%)	60 (51.7%)	0.052
Azathioprine	89 (7.5%)	16 (13.8%)	0.017
Mycophenolate	89 (7.5%)	14 (12.1%)	0.082
Cyclophosphamide	104 (8.8%)	14 (12.1%)	0.236
Ca channel antagonist	760 (64.0%)	87 (75.0%)	0.018
Topical vasodilator	93 (7.8%)	12 (10.3%)	0.343
Iloprost	150 (12.6%)	11 (9.5%)	0.324
Penicillamine	95 (8.0%)	13 (11.2%)	0.232
Rituximab	9 (0.8%)	2 (1.7%)	0.256^#^
Abatacept	1 (0.1%)	0 (0%)	> 0.999^#^
TNF alpha inhibitor	9 (0.8%)	2 (1.7%)	0.256^#^
Tocilizumab	4 (0.3%)	1 (0.9%)	0.373
Biologics	21 (1.8%)	5 (4.3%)	0.062

Values are given as number (%), mean ± SD or median (IQR)

*Abbreviations: ANA* anti nuclear antibodies, *ANCA* anti-neutrophil cytoplasmic antibodies, anti-phospholipid antibodies: any one or combination of anti-cardiolipin, anti-beta-2-glycoprotein and lupus anticoagulant, *anti-dsDNA* anti-double stranded deoxyribonucleic acid, *anti-RNP* ribonucleoprotein, *SIBO* small intestinal bacterial overgrowth, *SLE* systemic lupus erythematosus, *anti-Sm* anti-Smith

^^^Rank sum test

^#^Fisher’s exact test

Only 3 ANCA-positive patients had AAV (2.6% of the ANCA-positive cohort, 0.23% of the entire study cohort). One patient with AAV had biopsy-proven vasculitis with mononeuritis multiplex and anti-MPO antibodies. The second patient had p-ANCA-positive biopsy-proven crescentic glomerulonephritis, and the third patient had anti-MPO antibodies and biopsy-proven focal necrotising glomerulonephritis.

There was no statistically significant difference in gender, disease subtype, age of scleroderma onset, disease duration at recruitment, or duration of follow-up between ANCA-positive and ANCA-negative groups. There was a higher proportion of Asian patients in the ANCA-positive group (12.9% vs 3.7%, *p* = 0.001). ANCA-positive patients were less likely to have ever smoked (36.2% vs 50.3%, *p* = 0.004).

With respect to autoantibodies, ANCA-positive patients were more likely to be anti-Scl-70-positive (25.0% vs 12.8%, *p* < 0.001) and anti-Ro-positive (11.2% vs 6.2%, *p* = 0.040) than ANCA-negative patients. Anti-centromere antibodies were less common in the ANCA-positive group (29.3% vs 48.8%, *p* < 0.001).

Several clinical features were more common in the ANCA-positive group, including a significantly higher prevalence of ILD (44.8% vs 21.8%, *p* < 0.001), synovitis (54.3% vs 38.2%, *p* = 0.001), overlap features with another connective tissue disease (12.1% vs 5.2%, *p* = 0.003) and overlap features with Sjogren’s syndrome (4.3% vs 1.6%, *p* = 0.038). ANCA-positive patients were more likely to have had a pulmonary embolism (8.6% vs 3.0%, *p* = 0.002) and malignancy (26.7% vs 18.6%, *p* = 0.035). ANCA-positive patients were more likely to be hospitalised (49.1% vs 36.5%, *p* = 0.007).

The ANCA-positive group was more likely to have received azathioprine (13.8% vs 7.5%, *p* = 0.017) and calcium channel antagonists (75.0% vs 64.0%, *p* = 0.018). There was a trend towards increased use of prednisolone and biologics in ANCA-positive patients, but this did not reach statistical significance.

### Anti-MPO-positive vs anti-MPO-negative groups

A total of 13 patients were anti-MPO positive (11.2% of the ANCA-positive group). Univariable analyses comparing demographic characteristics between anti-MPO-positive and anti-MPO-negative patients are summarised in Table 3.

**Table 3:** Characteristics of anti-MPO-positive vs anti-MPO-negative patients and anti-PR3-positive vs anti-PR3-negative patients

	Anti-MPO negative *n* = 1289	Anti-MPO positive *n* = 13	*p*	Anti-PR3 negative *n* = 1284	Anti-PR3 positive *n* = 18	*p*
Female	1115 (86.5%)	9 (69.2%)	0.089^#^	1112 (86.6%)	12 (66.7%)	0.014
Race						
Caucasian	1166 (90.5%)	12 (92.3%)	0.622^#^	1164 (90.7%)	14 (77.8%)	0.024^#^
Asian	57 (4.4%)	1 (7.7%)		54 (4.2%)	4 (22.2%)	
Aboriginal-Islander	15 (1.2%)	0 (0%)		15 (1.2%)	0 (0%)	
Other	18 (1.4%)	0 (0%)		18 (1.4%)	0 (0%)	
Type of disease						
Limited	966 (74.9%)	8 (61.5%)	0.268	963 (75.0%)	11 (61.1%)	0.178
Diffuse	323 (25.1%)	5 (38.5%)		321 (25.0%)	7 (38.9%)	
Age of scleroderma onset, years	46.44 ± 14.27	42.23 ± 14.26	0.290	46.39 ± 14.29	46.86 ± 13.24	0.888
Age at recruitment, years	57.76 ± 12.52	53.07 ± 14.02	0.180	57.71 ± 12.56	58.02 ± 11.17	0.916
Disease duration at recruitment, years	8.31 (12.31)	13.07 (17.33)	0.924^^^	8.29 (13.30)	11.31 (16.86)	0.934^^^
Duration of follow-up, years	3.09 (4.50)	3.36 (3.48)	0.619^^^	3.09 (4.50)	3.97 (3.83)	0.640^^^
Ever smoked	633 (49.1%)	6 (46.2%)	0.832	634 (49.4%)	5 (27.8%)	0.069
Autoantibodies						
Anti-Scl-70	175 (13.6%)	5 (38.5%)	0.006	172 (13.4%)	8 (44.4%)	< 0.001
Anti-centromere	612 (47.6%)	1 (7.7%)	0.004^#^	608 (47.4%)	5 (27.8%)	0.092
ANA	1211 (93.9%)	10 (76.9%)	0.036^#^	1205 (93.8%)	16 (88.9%)	0.285^#^
Rheumatoid factor	335 (26.0%)	2 (15.4%)	0.532^#^	333 (25.9%)	4 (22.2%)	0.793^#^
Anti-Ro	87 (6.7%)	0 (0.0%)	> 0.999^#^	86 (6.7%)	1 (5.6%)	> 0.999^#^
Anti-La	20 (1.6%)	0 (0.0%)	> 0.999^#^	20 (1.6%)	0 (0%)	> 0.999^#^
Anti-RNP	26 (2.0%)	0 (0.0%)	> 0.999^#^	26 (2.0%)	0 (0%)	> 0.999^#^
Anti-dsDNA	41 (3.2%)	1 (7.7%)	0.385^#^	40 (3.1%)	2 (11.1%)	0.157^#^
Anti-Sm	8 (0.6%)	0 (0.0%)	> 0.999^#^	8 (0.6%)	0 (0.0%)	> 0.999^#^
Anti-Jo-1	7 (0.5%)	0 (0.0%)	> 0.999^#^	7 (0.5%)	0 (0.0%)	> 0.999^#^
Anti-PM/Scl	15 (1.2%)	0 (0.0%)	> 0.999^#^	15 (1.2%)	0 (0.0%)	> 0.999^#^
Anti phospholipid antibodies	302 (23.4%)	2 (15.4%)	> 0.999^#^	397 (30.9%)	7 (38.9%)	0.105
Anti-cardiolipin	259 (20.1%)	2 (15.4%)	> 0.999^#^	234 (18.2%)	7 (38.9%)	0.600^#^
Anti-beta 2 glycoprotein	93 (7.2%)	1 (7.7%)	0.539^#^	93 (7.2%)	1 (5.6%)	0.668^#^
Lupus anticoagulant	34 (2.6%)	1 (7.7%)	0.226^#^	35 (2.7%)	0 (0.0%)	> 0.999^#^
Anti-RNA polymerase	113 (8.8%)	3 (23.1%)	0.136^#^	116 (9.0%)	0 (0.0%)	0.149^#^
Clinical characteristics and complications						
Pulmonary arterial hypertension	121 (9.4%)	1 (7.7%)	> 0.999^#^	118 (9.2%)	4 (22.2%)	0.080^#^
Interstitial lung disease	305 (23.7%)	5 (38.5%)	0.213	301 (23.4%)	9 (50.0%)	0.009
Renal crisis	35 (2.7%)	0 (0.0%)	> 0.999^#^	35 (2.7%)	0 (0.0%)	> 0.999^#^
Digital ulcers	639 (49.6%)	6 (46.2%)	0.806	635 (49.5%)	10 (55.6%)	0.607
SIBO	37 (2.9%)	0 (0.0%)	> 0.999^#^	37 (2.9%)	0 (0.0%)	> 0.999^#^
GAVE	112 (8.7%)	0 (0.0%)	0.616^#^	112 (8.7%)	0 (0.0%)	0.393^#^
Oesophageal dysmotility	130 (10.1%)	1 (7.7%)	> 0.999^#^	130 (10.1%)	1 (5.6%)	> 0.999^#^
Oesphageal stricture	143 (11.1%)	3 (23.1%)	0.171^#^	145 (11.3%)	1 (5.6%)	0.711^#^
Synovitis	509 (39.5%)	7 (53.8%)	0.292	505 (39.3%)	11 (61.1%)	0.061
Malignancy	250 (19.6%)	2 (15.4%)	> 0.999^#^	249 (19.4%)	3 (16.7%)	> 0.999^#^
Hospitalised	484 (37.5%)	6 (46.2%)	0.524	481 (37.5%)	9 (50.0%)	0.276
Deep vein thrombosis	89 (6.9%)	1 (7.7%)	0.608^#^	89 (6.9%)	1 (5.6%)	> 0.999^#^
Pulmonary embolus	44 (3.4%)	1 (7.7%)	0.368^#^	42 (3.3%)	3 (16.7%)	0.022^#^
Overlap features with another connective tissue disease	74 (5.7%)	2 (15.4%)	0.173^#^	74 (5.8%)	2 (11.1%)	0.283^#^
Overlap syndrome with rheumatoid arthritis	25 (1.9%)	2 (15.4%)	0.028^#^	25 (1.9%)	2 (11.1%)	0.052^#^
Overlap syndrome with polymyositis	13 (1.0%)	0 (0.0%)	> 0.999^#^	13 (1.0%)	0 (0.0%)	> 0.999^#^
Overlap syndrome with Sjogren’s	24 (1.9%)	0 (0.0%)	> 0.999^#^	24 (1.9%)	0 (0.0%)	> 0.999^#^
Overlap syndrome with SLE	12 (0.9%)	0 (0.0%)	> 0.999^#^	12 (0.9%)	0 (0.0%)	> 0.999^#^
Overlap syndrome with dermatomyositis	1 (0.1%)	0 (0.0%)	> 0.999^#^	1 (0.1%)	0 (0.0%)	> 0.999^#^
Treatments						
Prednisolone	570 (44.2%)	9 (69.2%)	0.093^#^	568 (44.2%)	11 (61.1%)	0.153
Azathioprine	101 (7.8%)	3 (23.1%)	0.079^#^	101 (7.9%)	3 (16.7%)	0.169^#^
Mycophenolate	100 (7.8%)	2 (15.4%)	0.271^#^	98 (7.6%)	4 (22.2%)	0.046^#^
Cyclophosphamide	113 (8.8%)	4 (30.8%)	0.023^#^	113 (8.8%)	4 (22.2%)	0.071^#^
Calcium channel antagonist	836 (64.9%)	10 (76.9%)	0.364^#^	833 (64.9%)	13 (72.2%)	0.516
Topical vasodilator	103 (8.0%)	2 (15.4%)	0.283^#^	103 (8.0%)	2 (11.1%)	0.651^#^
Iloprost	160 (12.4%)	1 (7.7%)	> 0.999^#^	159 (12.4%)	2 (11.1%)	> 0.999^#^
Operation for peripheral vascular disease	14 (1.1%)	0 (0.0%)	> 0.999^#^	14 (1.1%)	0 (0%)	> 0.999^#^
Penicillamine	105 (8.1%)	3 (23.1%)	0.086^#^	105 (8.2%)	3 (16.7%)	0.183^#^
Rituximab	10 (0.8%)	1 (7.7%)	0.105^#^	10 (0.8%)	1 (5.6%)	0.142^#^
Abatacept	1 (0.1%)	0 (0.0%)	> 0.999^#^	1 (0.1%)	0 (0%)	> 0.999^#^
TNF alpha inhibitor	11 (0.9%)	0 (0.0%)	> 0.999^#^	11 (0.9%)	0 (0%)	> 0.999^#^
Tocilizumab	5 (0.4%)	0 (0.0%)	> 0.999	5 (0.4%)	0 (0%)	> 0.999
Biologics ever	25 (1.9%)	1 (7.7%)	0.232^#^	25 (1.5%)	1 (5.6%)	0.306^#^

Values are given as number (%), mean ± SD or median (IQR)

*Abbreviations: ANA* anti nuclear antibodies, *ANCA* anti-neutrophil cytoplasmic antibodies, anti-phospholipid antibodies: any one or combination of anti-cardiolipin, anti-beta-2-glycoprotein and lupus anticoagulant, *anti-dsDNA* anti-double stranded deoxyribonucleic acid, *anti-RNP* ribonucleoprotein, *SIBO* small intestinal bacterial overgrowth, *SLE* systemic lupus erythematosus, *anti-Sm* anti-Smith

^Rank sum test

^#^Fisher’s exact test

Demographic characteristics including gender, race, disease subtype and age of scleroderma onset were similar between anti-MPO-positive and anti-MPO-negative groups. For autoantibodies, anti-MPO-positive patients were more likely to be anti-Scl-70 positive (38.5% vs 13.6%, *p* = 0.006) and were less likely to be ANA positive (76.9% vs 93.9%, *p* = 0.036) than the anti-MPO-negative group. Anti-MPO-positive patients were more likely to have an overlap syndrome with rheumatoid arthritis (15.4% vs 1.9%, *p* = 0.028). There was a trend towards a higher prevalence of ILD in the anti-MPO-positive group, but this did not reach statistical significance (38.5% vs 23.7%, *p* = 0.213). There were no other statistically significant differences in disease features between anti-MPO-positive and negative groups. Anti-MPO-positive patients were more likely to receive cyclophosphamide (30.8% vs 8.8%, *p* = 0.023).

### Anti-PR3-positive vs anti-PR3-negative groups

A total of 18 patients were anti-PR3 positive (13.8% of the ANCA-positive cohort). Univariable analyses comparing demographic characteristics between anti-PR3-positive and anti-PR3-negative patients are summarised in Table 3.

A higher proportion of male patients (33.3% vs 13.4%, *p* = 0.014) and Asian patients (22.2% vs 4.2%, *p* = 0.024) were found in the anti-PR3 positive compared to anti-PR3-negative groups. Other demographic characteristics including disease subtype, age of scleroderma onset, age at recruitment and duration of follow-up were similar between the two groups.

Anti-PR3-positive patients were more likely to be anti-Scl-70 positive than the anti-PR3-negative group (44.4% vs 13.4%, *p* < 0.001). A significantly higher prevalence of ILD (50.0% vs 23.4%, *p* = 0.009) and pulmonary embolism (16.7% vs 3.3%, *p* = 0.022) was found in the anti-PR3-positive group. There was a higher prevalence of overlap syndrome with rheumatoid arthritis (61.1% vs 39.3%, *p* = 0.061) and synovitis (11.1% vs 1.9%, *p* = 0.052) in the anti-PR3-positive group, but this did not reach statistical significance. A higher proportion of anti-PR3-positive patients had received mycophenolate compared to the anti-PR3-negative group (22.2% vs 7.6%, *p* = 0.046).

### Multivariable correlates of ANCA

Variables included in a multivariable model of associations of ANCA were based on those statistically significant in univariable analysis. We excluded “overlap syndrome with Sjogren’s” due to multicollinearity with other variables. The final multivariable model included ILD (OR 2.85, 95% CI 1.92–4.23, *p* = <0.0001), PE (OR 2.82, 95% CI 1.34–5.95, *p* = 0.007), overlap syndrome (OR 2.25, 95% CI 1.19–4.25, *p* = 0.013) and synovitis (OR 1.73, 95% CI 1.16–2.56, *p* = 0.007) (Table 4).

**Table 4:** Multivariable logistic regression analysis for clinical correlates of ANCA

Clinical characteristic	OR	95% CI	*p*
ILD	2.85	1.91–4.23	<0.0001
Pulmonary embolism	2.82	1.34–5.95	0.007
Overlap syndrome	2.25	1.19–4.25	0.013
Synovitis	1.72	1.16–2.56	0.007

*Abbreviations: ILD* interstitial lung disease

### Multivariable analysis of the relationship between ANCA and interstitial lung disease

In multivariable regression analysis, ANCA was independently associated with ILD (OR 2.63, 95% CI 1.72–4.0, *p* < 0.001) after taking into account anti-Scl-70 antibodies.

### Multivariable analysis of the relationship between ANCA and pulmonary embolism

In multivariable regression analysis, ANCA was independently associated with pulmonary embolism even after taking into account the presence of anti-phospholipid antibodies (OR 3.11, 95% CI 1.49–6.48, *p* = 0.003).

### Survival analysis

**Fig. 1:**
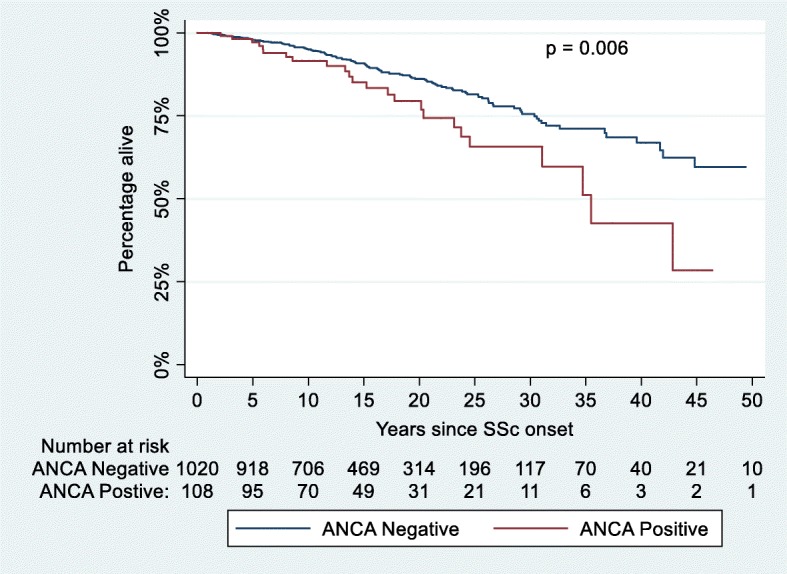
Kaplan-Meier curve comparing survival in the ANCA-positive and ANCA-negative patients. SSc onset refers to the date of onset of first non-Raynaud’s manifestation. *Abbreviations*: ANCA anti-neutrophil cytoplasmic antibodies, SSc systemic sclerosis

Kaplan-Meier analysis comparing survival in ANCA-positive and ANCA-negative patients (Fig. 1) revealed ANCA-positive patients had significantly increased mortality (*p* = 0.006). ANCA remained associated with higher mortality after adjusting for age at SSc onset and sex in Cox regression analysis (HR 1.622, 95% CI 1.04–2.54, *p* = 0.034).

## Discussion

The prevalence of ANCA in this large SSc cohort was 8.9%, which is within the range of 0–12% reported in previous studies [7, 10, 11]. Previous studies have found anti-MPO to be the predominant ANCA subtype in SSc. Comparatively, there was a higher prevalence of anti-PR3 in our cohort (15.5% of ANCA-positive patients) rather than anti-MPO (11.2% of ANCA-positive patients).

Given the known association between penicillamine therapy and the development of ANCA antibodies [10, 16–19], it is notable that in this modern era cohort, relatively few patients (8.3%) had received penicillamine. In addition, there was no significant difference in exposure to penicillamine between ANCA positive and ANCA negative, anti-MPO positive and negative, and anti-PR3 positive and negative groups. This suggests that exposure to penicillamine is not responsible for the difference in ANCA status between these groups.

Overlap syndrome with AAV in our cohort was rare, with only 3 cases of AAV (0.23% of the entire cohort, 2.6% of ANCA-positive patients). This low prevalence of AAV is comparable to a previous study of 2200 SSc patients, which reported a prevalence of AAV of only 0.40% [12]. The reason for the significant majority of ANCA-positive SSc patients not manifesting vasculitis is unclear. Theories for this discordance are that the presence of ANCA is an epiphenomenon of SSc, that ANCA present in SSc may have poor affinity and avidity for the epitope and are therefore less pathogenic, that there may be false positive ANCA results or that ANCA has been detected prior to the future onset of AAV [8].

There were several differences in demographics between ANCA-positive and ANCA-negative patients. ANCA-positive patients were more likely to be Asian, and this difference was also observed in the anti-PR3-positive group compared to the anti-PR3-negative cohort. A higher proportion of male patients were found in the anti-PR3-positive group, which is notable given that SSc male patients have been shown to have a higher prevalence of ILD and increased mortality [26].

Limited disease predominated in our entire cohort (74.8%) and in both the ANCA-positive and ANCA-negative groups. Despite a predominance of lcSSc in all groups, there was a significantly higher prevalence of anti-Scl-70 in the ANCA-positive group compared to the ANCA-negative group, the anti-MPO-positive group compared to the anti-MPO-negative group, and the anti-PR3-positive group compared to the anti-PR3-negative group. Accordingly, as these antibodies are mutually exclusive, there was also a lower prevalence of anti-centromere antibodies in the ANCA-positive, anti-MPO-positive and anti-PR3-positive groups. There was a significantly lower prevalence of anti-nuclear antibody in the anti-MPO-positive group.

There was a significantly higher prevalence of ILD in the ANCA positive compared to ANCA-negative group and also the anti-PR3-positive group compared to the anti-PR3-negative group. Importantly, in multivariable analysis, the relationship between ANCA and increased prevalence of ILD was independent of anti-Scl-70. To our knowledge, this is the first study to find an independent association of ANCA and ILD in SSc patients in the absence of comorbid AAV. An association between anti-MPO antibodies and ILD in patients with rheumatoid arthritis without comorbid AAV has been reported in a small retrospective analysis, with 3 of 12 (25%) patients with anti-MPO antibodies manifesting ILD compared to 0 of 85 patients without anti-MPO antibodies [27]. The authors suggested subclinical vasculitis might have led to pulmonary fibrosis in these 3 patients [27]. For patients with SSc/AAV overlap, it is estimated that the prevalence of ILD is around 80%, which is far higher than that expected for each condition individually [12, 28]. It is suggested that a high susceptibility to ILD in patients with AAV/SSc overlap may be related to an inflammatory insult or due to oxidative stress caused by ANCA in patients with a predisposition to ILD due to their SSc [12, 28]. In our study, the underlying cause of the association between ANCA and increased prevalence of ILD in SSc is unclear. Our findings may suggest a pathological role of ANCA in the development of ILD in SSc, perhaps mediated by subclinical vasculopathy; however, it is also possible that ILD itself induces the development of ANCA antibodies. The temporal relationship between the development of ILD and the emergence of ANCA was not explored in this study.

Our study demonstrates an association between ANCA and PE, and the increased prevalence of PE was also observed in the anti-PR3-positive compared to the anti-PR3-negative group. Notably, in multivariable analysis, this association was independent of the presence of anti-phospholipid antibodies. Patients with AAV are known to have an increased prevalence of venous thromboembolism, particularly when AAV is active; however, the underlying cause for this increased risk is unknown [29]. Our findings suggest that ANCA may have a prothrombotic effect even in the absence of AAV.

Previous studies have suggested that patients with comorbid SSc and AAV have a higher prevalence of overlap syndromes with other connective tissue diseases [14]. Our study demonstrates that even in the absence of AAV, ANCA-positive SSc patients have a higher prevalence of overlap features with another connective tissue disease and specifically a higher prevalence of overlap with Sjogren’s syndrome. With respect to treatments, ANCA-positive patients were more likely to receive azathioprine and anti-PR3-positive patients were more likely to receive mycophenolate. These findings may be attributable to the increased prevalence of ILD in these groups. Finally, ANCA-positive status was associated with a 1.6 fold increased hazard of mortality identifying a subset of patients with worse prognosis. While cause-specific mortality was not evaluated, the increased mortality associated with ANCA may be related to ILD, thromboembolic complications and multi-organ involvement seen in overlap SSc.

To our knowledge, this is the largest study investigating the clinical associations of ANCA in SSc published to date. This study is also strengthened by a comprehensive evaluation of associations with disease features and mortality using prospectively collected data. However, the results need to be interpreted within the limitations of the study design. We were unable to check for all possible confounders such as thyroid and liver disease that are known to be associated with ANCA, as information regarding these conditions is not collected in the cohort study. Whilst data are collected prospectively at each annual study visit, this analysis was performed retrospectively, and some data were missing for some patients. In particular, only 67.5% of patients had anti-RNA polymerase III antibodies tested. However, for the remainder of variables, the percentage missing data was below 10%. Even though the study included a large number of patients, the overall prevalence of each of anti-PR3 and anti-MPO was low, limiting the power of the statistical analyses to draw robust conclusions. Lack of data on serial measurement of anti-MPO and anti-PR3 titre is also a limitation, as is lack of confirmation of all ENA and anti-dsDNA testing using immunoprecipitation or Western blot, and the Farr radioimmunoassay, respectively.

## Conclusions

This study reveals a significant association between ANCA in SSc and ILD, pulmonary embolism, synovitis and overlap syndrome with other connective tissue diseases. Patients who are ANCA positive have increased mortality, independent of sex or age at diagnosis. These findings suggest that ANCA should be tested at baseline in SSc patients as it is associated with a worse prognosis and necessitates vigilant monitoring and follow-up.

## Abbreviations

AAV: Anti-neutrophil cytoplasmic antibody (ANCA)-associated vasculitis
ACR: American College of Rheumatology
ANA: Anti-nuclear antibodies
ANCA: Anti-neutrophil cytoplasmic antibodies
ASCS: Australian Scleroderma Cohort Study
c-ANCA: Cytoplasmic ANCA
dcSSc: Diffuse cutaneous systemic sclerosis
DM: Dermatomyositis
dsDNA: Anti-double-stranded deoxyribonucleic acid
ELISA: Enzyme-linked immunosorbent assay
IIF: Indirect immunofluorescence
ILD: Interstitial lung disease
lcSSc: Limited cutaneous systemic sclerosis
MPO: Myeloperoxidase
PAH: Pulmonary arterial hypertension
p-ANCA: Perinuclear ANCA
PE: Pulmonary embolism
PM: Polymyositis
PR3: Proteinase 3
RA: Rheumatoid arthritis
RNP: Ribonucleoprotein
SD: Standard deviation
SLE: Systemic lupus erythematosus
Sm: Anti-Smith
SS: Sjogren’s syndrome
SSc: Systemic sclerosis
